# Trace level detection of NH_3_ at room temperature using Cd-ZnFe_2_O_4_ thin films

**DOI:** 10.1016/j.isci.2025.114271

**Published:** 2025-11-29

**Authors:** Ravikumar Thangavel, Kalainathan Sivaperuman, Logu Thirumalaisamy, Christina Josephine Malathi A, Saravanan Pandiaraj, Maha Alruwaili, Nadyah Alanazi, Abdullah N. Alodhayb, R. Ramesh, Chamil Abeykoon, Andrews Nirmala Grace

**Affiliations:** 1 Centre for Nanotechnology Research, Vellore Institute of Technology, Vellore 632014, India; 2 Department of Physics, G.T.N Arts College (Affiliated to Madurai Kamaraj University), Dindigul, Tamil Nadu 624005, India; 3 Department of Communication Engineering, School of Electronics Engineering (SENSE), Vellore Institute of Technology, Vellore, Tamil Nadu 632014, India; 4 Department of Self-Development Skills, King Saud University, Riyadh 11451, Saudi Arabia; 5 Department of Physics, College of Science, Hafar Albatin UHB University, Hafar Albatin 31991, Saudi Arabia; 6 Department of Physics and Astronomy, College of Science, King Saud University, Riyadh 11451, Saudi Arabia; 7 King Salman Center for Disability Research, Riyadh 11614, Saudi Arabia; 8 Department of Physics, Periyar University, Salem, Tamil Nadu 636 011, India; 9 Northwest Composites Centre, Aerospace Research Institute, and Department of Materials, Faculty of Science and Engineering, The University of Manchester, Oxford Road, Manchester M13 9PL, UK

**Keywords:** materials science, materials processing, materials application

## Abstract

This study focuses on developing economical and efficient ammonia (NH_3_) gas sensors capable of detecting low concentrations at room temperature. Cd-doped ZnFe_2_O_4_ (Cd_x_Zn_1-x_Fe_2_O_4_, x = 0, 0.1,0.3, and 0.5) thin films were deposited via spray pyrolysis, showing significantly enhanced sensing performance compared to undoped ZnFe_2_O_4_. The Cd_0.5_Zn_0.5_Fe_2_O_4_ (ZFCD5) film demonstrated the best response (∼8) at 1 ppm NH_3_, with fast response (105 s) and recovery (54 s) times, a sensitivity of 10.07 ppm^-1^, repeatability, selectivity, and more stability over 6 weeks. The improved sensing is attributed to the angular-rod-like morphology that increases active sites and enhances charge transfer. Cd incorporation effectively boosts defect density and adsorption-desorption efficiency, resulting in a 10-fold higher response than the undoped film (∼0.8). The findings highlight the potential of Cd-doped ZnFe_2_O_4_ thin films as promising, room temperature NH_3_ sensors for industrial, environmental, and safety applications, also supporting safer environments for individuals with disabilities.

## Highlights

• Cd-ZnFe_2_O_4_ film prepared via the spray pyrolysis method
• ZFCD5 film sensor response of ∼8 at low concentration 1 ppm NH_3_
• Proposed ZFCD5 film moderate response (105 s) and faster recovery (54 s) times
• ZFCD5 film exhibits good sensitivity (10.07 ppm^-1^), repeatability, and stability

## Graphical abstract

**Figure:**
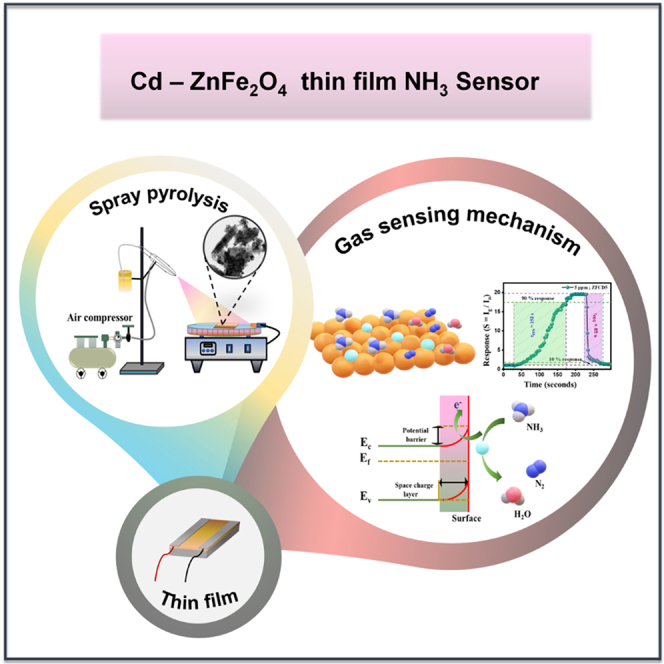

## Introduction

Ammonia (NH_3_) is extensively used in chemical manufacturing, refrigeration, and agriculture.^1^^,^^2^ Despite its utility, NH_3_ leaks pose serious health and environmental risks due to its toxic and corrosive nature.^3^ Exposure to high concentrations can cause respiratory issues, eye irritation, and even fatal consequences. Similarly, NH_3_ is used in the agricultural sector, with uncontrolled NH_3_ emissions contributing to soil acidification and air pollution. This may harm human health and environment.^4^ According to the Occupational Safety and Health Administration database, the permissible exposure limit for NH_3_ is 50 ppm (parts per million), averaged over an 8-h workday.^5^ So, an efficient and reliable NH_3_ sensor is critical for ensuring workplace protection, atmosphere protection, and regulatory compliance. Advanced gas sensors are crucial in real-time monitoring and early detection of NH_3_ leaks, preventing potential hazards and ensuring operational efficiency. In recent decades, the synthesis and properties of transition metal ferrites (TMFs) with the structural formula MFe_2_O_4_ (M = Zn, Ni, Co, and Mn) have been extensively studied due to their technological significance.^6^ Many TMFs play a vital role in semiconductor devices because of their outstanding electrical, optical, and magnetic properties. Among these, ZnFe_2_O_4_ has garnered significant attention for its potential applications in photocatalysis,^7^ ferrofluids,^8^ magnetic data storage,^9^ and medical imaging.^10^ Apart from these applications, ZnFe_2_O_4_ has emerged as a potential chemiresistive material for the detection of NH_3_, trimethylamine, H_2_S, CO_2_, SO_2_, and volatile organic compounds (VOCs) at very low working temperatures, attributable to its small band gap (∼1.9 eV), excellent chemical stability, and adjustable surface chemistry. Recent research indicates that nanostructured ZnFe_2_O_4_ (including nanoparticles, nanorods, and hollow spheres) has enhanced sensitivity and reduced response and recovery periods.^11^^,^^12^^,^^13^^,^^14^^,^^15^^,^^16^^,^^17^^,^^18^^,^^19^ ZnFe_2_O_4_ is an inorganic compound characterized by its environmental friendliness, low toxicity, and abundant availability of natural resources.^20^^,^^21^ ZnFe_2_O_4_ is a typical spinel, where Zn^2+^ resides in tetrahedral sites, and Fe^3+^/Fe^2+^ are located in octahedral sites. This cation configuration facilitates electron transfer between Fe^2+^ and Fe^3+^, hence improving charge transport.^22^ The spinel lattice accommodates a significant concentration of oxygen vacancies, which serve as active adsorption sites for electron-donating or electron-withdrawing gases, thereby enhancing adsorption-desorption dynamics.^23^ Although ZnFe_2_O_4_ based sensors have shown better gas sensing performance, they still suffer from limitations such as low sensor response and limited detection of low gas concentrations. Doping is an effective strategy to overcome these challenges and enhance gas sensing performance. In particular, cadmium (Cd^2+^) doping in ZnFe_2_O_4_ has been explored to modify the material’s electrical and structural properties. Cd^2+^ ionic radius 0.95 Å is quite bigger than Zn^2+^ ionic radius 0.74 Å; therefore, its integration into the Zn sublattice results in lattice expansion, alteration of defect density, and elevation of oxygen vacancy concentration.^24^ These modifications augment charge-carrier density and elevate surface reactivity toward electron-donating analytes such as NH_3_. Cd^2+^ incorporates into the ZnFe_2_O_4_ lattice, reducing the band gap, hence enhancing electron excitation and expediting response and recovery kinetics.^24^^,^^25^ Several studies have investigated Cd-doped ZnFe_2_O_4_ for different applications. For example, K. N. Harish et al. studied Cd-substituted ZnFe_2_O_4_ for photocatalytic applications and found that Cd substitution shifted the absorption edge of ZnFe_2_O_4_ toward the red region, thereby increasing its visible light absorption.^26^ Similarly, using the co-precipitation method, Aamir Mahmood et al. synthesized Cd-doped ZnFe_2_O_4_ nanoparticles. Their results showed that Cd doping increased lattice parameters and nanoparticle size, altered bandgap energies and photoluminescence emission, and influenced magnetic properties by reducing magnetic saturation while increasing coercivity and magneto-crystalline anisotropies.^27^ Zn_1-x_Cd_x_Fe_2_O_4_ powders were produced by Chu Xianfeng et al. and analyzed utilizing co-precipitation and solid-state reaction techniques. The introduction of Cd^2+^ ions into the ZnFe_2_O_4_ lattice resulted in an augmentation in crystal size and lattice properties while maintaining the spinel structure. Significantly, Cd doping improved the sensor’s sensitivity from 44 to 1000 ppm C_2_H_5_OH, with response (10 s) and recovery (20 s) times.^28^ Hani Korek et al. prepared and studied Cd_0.5_Zn_0.5_Sm_x_Fe_2-x_O_4_ nanoparticles using a wet chemical co-precipitation technique. The synthesized particles demonstrated a favorable response (S = 700 at 385 °C) and selectivity for LPG. The improved gas-sensing sensitivity is due to the synergistic impact of Cd and Sm inclusion, which reduces crystallite size and generates oxygen vacancies.^29^ These findings indicate that the incorporation of Cd into the ZnFe_2_O_4_ lattice reduces the band-gap energy, reduces the crystallite size, generates oxygen vacancies, and modifies the lattice structure. These alterations improve the material’s gas-sensing capabilities. Moreover, Cd-doped ZnFe_2_O_4_ has been tried for NH_3_ gas sensing applications. To the best of our knowledge, no prior studies have focused explicitly on sensing NH_3_ gas at trace level concentration and at room temperature using Cd-doped ZnFe_2_O_4_ sensors. Many deposition techniques are used to deposit zinc ferrite thin film.^30^^,^^31^ Among the deposition techniques, chemical spray pyrolysis is one of the solid methods to produce large-scale, cost-effective thin films. Moreover, the properties of the film can be reformed by changing the deposition conditions, and there is no need for a vacuum environment.^32^^,^^33^^,^^34^^,^^35^

The main objective of this work is to improve the physical, topographical, and gas sensing characteristics of Zn_1-x_Cd_x_Fe_2_O_4_ (x = 0, 0.1, 0.3, and 0.5) films synthesized via the spray pyrolysis method for detecting low concentrations of NH_3_ at ambient temperature. This study focuses on improving the NH_3_ sensing performance of Zn_1-x_Cd_x_Fe_2_O_4_ films, especially at room temperature. In our previous study, ZnFe_2_O_4_ thin films were coated at different substrate temperatures via spray pyrolysis, and the 225 °C substrate temperature coated films exhibited a response of 8 toward NH_3_ at 25 ppm.^36^ However, the sensor could not detect NH_3_ at low concentrations, indicating a limitation in sensitivity. To address this issue, Cd doping is introduced to enhance the sensing capabilities of ZnFe_2_O_4_ thin film. This research aims to bridge the gap by investigating the gas sensing performance, low concentration detection, and sensitivity of Cd-doped ZnFe_2_O_4_ thin films toward NH_3_. These findings provide valuable insights into the material’s potential as an efficient NH_3_ sensor. The improved sensing characteristics of these doped films also open up different possibilities for detecting various hazardous gases in industrial and environmental monitoring applications. Furthermore, reliable NH_3_ detection is especially important for people who may have limited ability to sense or react to gas leaks, such as individuals with respiratory conditions, sensory impairments, or other disabilities. Improving sensor accessibility and ease of use can help protect vulnerable populations, making workplaces and homes safer.

## Results

### Crystal structure, morphology, optical, and electrical properties

**Figure 1:**
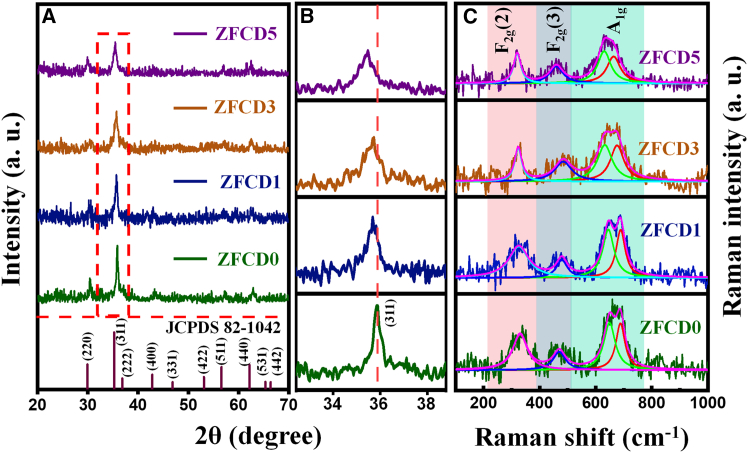
Structural characterization of the ZFCD0-ZFCD5 film (A) XRD pattern of undoped and doped ZnFe_2_O_4_ films. (B) Amplified XRD pattern illustrates the shift of the peak of maximum intensity. (C) Raman spectra of ZFCD0-ZFCD5 films.

The structural properties of the deposited doped and undoped ZnFe_2_O_4_ thin films were analyzed using XRD. The XRD patterns of the deposited film are given in Figure 1A. From the diffraction patterns, the (220), (311), (222), (400), (422), (511), and (440) reflection planes confirm the formation of a cubic spinel structure with the Fd3m space group. All diffraction peaks were indexed, and the relative intensities of the peaks match well with the standard ZnFe_2_O_4_ powder diffraction data (JCPDS) card No. 82-1042. There is no secondary peak present in the diffraction pattern. The sharp and intense peaks indicated good crystallinity of the deposited film. With increased Cd dopant concentration, a noticeable relocation in the XRD peaks in the direction of lower angles was observed. The (311) peak shifts from 35.87° (undoped) to 35.48° (ZFCD5). This shift can be attributed to the substitution of Zn^2+^ ions (ionic radius ∼0.74 Å) by Cd^2+^ ions (ionic radius ∼0.95 Å) in the ZnFe_2_O_4_ lattice.^27^ Further, as demonstrated in the partially extended XRD pattern (Figure 1B), the larger ionic radius of Cd^2+^ causes lattice expansion, leading to an increase in the interplanar spacing (d-spacing) according to Bragg’s law. As a result, the diffraction peaks move toward lower angles. A similar lower angle shift was observed by Aamir Mahmood et al. when Cd was incorporated into the ZnFe_2_O_4_ material.^27^ The lattice constant was calculated by using the formula

(Equation 1) $$a=d_{\text{hkl}}\sqrt{h^{2}+k^{2}+l^{2}}$$

where d_hkl_ - d spacing of the hkl plane. The lattice constant value increases linearly with an increase in the Cd concentration (8.4103–8.4416 Å). From the XRD pattern, the crystallite size was measured utilizing the Debye-Scherrer formula.^37^ The observed crystallite size decreased with increased Cd^2+^ concentration (Table 1). The dislocation density was calculated by using Equation 2.

(Equation 2) $$\delta =\frac{1}{D^{2}}$$

where *δ* – dislocation density and D – crystallite size.

**Table 1:** Lattice constant, cell volume, crystallite size, and dislocation density of doped and undoped ZnFe_2_O_4_ films

Film code	Lattice constant (Å)	Cell volume (Å)	Crystallite size (nm)	Dislocation density x 10^−3^ (nm^−2^)
ZFCD0	8.4103	594	27	1.3
ZFCD1	8.4216	597	25	1.6
ZFCD3	8.4355	600	24	1.7
ZFCD5	8.4416	601	22	1.9

This structural modification due to Cd doping also slightly affects the crystallite size and dislocation density, influencing the material’s gas-sensing properties by altering charge transport mechanisms and surface reactivity. Higher dislocation density can present more active sites on the material surface. So, oxygen molecules can easily interact with the material and promote adsorption and desorption. The lattice constant, crystallite size, cell volume, and dislocation density values are given in Table 1.

Raman spectroscopy analysis was conducted to further confirm the structural integrity and vibrational modes of the ZnFe_2_O_4_ and Cd-doped ZnFe_2_O_4_ thin films. The spectrum of pure ZnFe_2_O_4_ has four active phonon modes at around 335, 480, 654, and 690 cm^−1^, characteristic of the spinel ferrite structure. These modes are parallel to the F_2g_ (2), F_2g_ (3), A_1g_ (2), and A_1g_ (1) modes. These four Raman active modes establish the deposited film’s spinel structure with the space group of Fd3m. Figure 1C presents the Raman peaks acquired for all the films. The active F_2g_ (2) mode above ∼300 cm^−1^ is associated with the motion of oxygen and the opposite movement of the cations. In contrast, F_2g_ (3) above ∼400 cm^−1^ is linked to the stretching vibration of oxygen in tetrahedral sites, while A_1g_ at 600 cm^−1^ is related to the phonon stretching vibration within the octahedral lattice sites. As the concentration of Cd^2+^ rises, the intensities of the F_2g_ (2), F_2g_ (3), and A_1g_ modes diminish and shift toward the lower wavenumber. The shift in the peaks of these four active modes may be dependent upon the redistribution of cations among the various sites within the spinel ferrite compound structure, influenced by the concentration of Cd ions.^24^ The shifts toward lower wavenumbers are attributed to the increased lattice strain and mass effect introduced by the Cd^2+^ ions, which alter the local vibrational environment of the ZnFe_2_O_4_ lattice.^38^ Consequently, the Raman spectra provided validation for the space group and the incorporation of Cd within the zinc ferrite structure. The Raman spectra of Cd-doped ZnFe_2_O_4_ film were examined, which conformed to a least-squares Lorentzian line structure. Figure 1C illustrates the optimal concordance between the experimental data curve and the theoretical data through the Lorentz fit. The active Raman modes over 600 cm^−1^ predominantly maintain the symmetrical mobility of oxygen ions at tetrahedral sites within the spinel lattice; hence, the modes at 640 and 690 cm^−1^ may be ascribed to the division of A_1g_ into A_1g_(2) and A_1g_(1) symmetries.^24^^,^^39^ This phenomenon can be ascribed to the random occupancy of tetrahedral sites by Zn^2+^ and Fe^3+^ cations, likely resulting in minor variations in frequencies, hence indicating the emergence of the observed double vibrational peaks for A_1g_. The A_1g_ (2) Raman mode pertains to Zn-O and Fe-O bonds, whereas the A_1g_(1) mode signifies the interaction of Fe-O bonds at tetrahedral locations.^6^^,^^40^ The two modes at ∼640 and 690 cm^−1^ correspond to the A_1g_ mode. The increase in Cd^2+^ ion concentration in zinc ferrite, along with cation redistribution, resulted in a rise in the A_1g_ mode peak intensity ratio between A_1g_(2) and A_1g_(1) (from 0.98 to 1.25), indicating a relative augmentation of tetrahedral site vibrations.^41^ Moreover, the spectra aligned seamlessly with the data reported in earlier studies.^42^^,^^43^ Additionally, the broadening of the peaks indicated increased structural disorder, suggesting slight modifications in the bonding environment.

**Figure 2:**
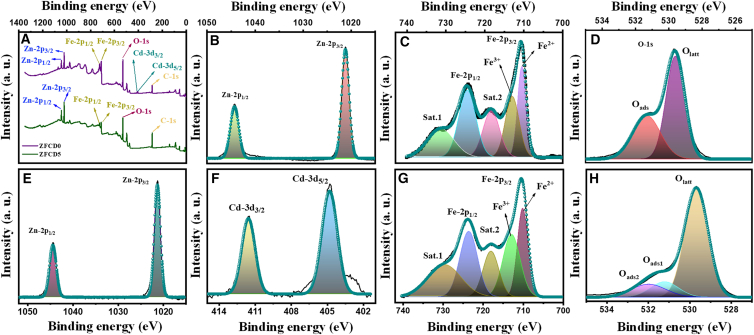
Chemical composition of ZFCD0 and ZFCD5 films (A) Full XPS survey spectra of ZFCD0 and ZFCD5 films. (B–D) XPS spectra of (B) Zn 2p, (C) Fe 2p, and (D) O 1s for the ZFCD0 film. (E–H) XPS spectra of (E) Zn 2p, (F) Cd 3d, (G) Fe 2p, and (H) O 1s for the ZFCD5 film.

XPS analysis was employed to identify the chemical composition of ZnFe_2_O_4_ film and ascertain the electronic valence states of the constituent components in this structure. The complete survey XPS spectra of ZFCD0 and ZFCD5 films are presented in Figure 2A, displaying the corresponding peaks for the elements C, Zn, Fe, O, and Cd. Two principal peaks with binding energies of 1044.32 eV and 1021.21 eV, as well as 1044.26 eV and 1021.12 eV, corresponding to Zn-2p_1/2_ and Zn-2p_3/2_, respectively, were seen in the Zn-2p spectra of the films ZFCD0 and ZFCD5, as illustrated in Figures 2B and 2E. The energy separation between Zn-2p_1/2_ and Zn-2p_3/2_ is 23.11 eV, suggesting the existence of Zn^2+^ in ZnFe_2_O_4_. The little shift in binding energies (1044.32–1044.26 eV) and the reduction in peak intensity may be ascribed to the greater radius of Cd^2+^ ions compared to Zn^2+^ ions.^44^ Figure 2F illustrates the two principal peaks at 404.80 and 411.52 eV, which are indicative of the spin orbitals 3d_5/2_ and 3d_3/2_, respectively, therefore affirming the existence of cadmium ions in the divalent form.^44^^,^^45^ The Fe-2p spectra of ZFCD0 and ZFCD5 films are presented in Figures 2C and 2G, with Fe-2p_3/2_ and Fe-2p_1/2_ peaks at binding energies of 710.52 eV, 724.48 eV, and 710.14 eV, 723.65 eV, respectively. The Fe-2p_3/2_ spectrum could be deconvoluted into two peaks with binding energies mostly centered at 710.52 eV and 713 eV, as well as 710.14 eV and 712.96 eV, attributable to the presence of Fe^2+^/Fe^3+^sites.^22^^,^^46^ The two satellite peaks at 718.33 and 731 eV correspond to the principal spin orbitals Fe-2p_3/2_ and Fe-2p_1/2_, respectively, while the additional shake-up signals at 712.96 and 723.65 eV are associated with the presence of Fe^3+^ in the tetrahedral sites. Figures 2D and 2H illustrates the O-1s spectra of ZFCD0 and ZFCD5. Two peaks, O_latt_ and O_ads_, are identified at 529.57 eV and 531.29 eV, respectively, for the ZFCD0 film. ZFCO5 films exhibit three peaks, O_latt_, O_ads1_, and O_ads2_, identified at 529.69, 531.19, and 532.02 eV, respectively, corresponding to the broad asymmetric curves observed in the spectra. The standard lattice oxygen on the surface of ferrites is accountable for the O_latt_ peak. In contrast, the O_ads1_ signal may be associated with O_2_ ions situated in matrix regions deficient in oxygen.^46^^,^^47^ A link exists between the intensity of this component and the concentration of defects related to oxygen vacancies.^48^ These defects are crucial for gas sensing since they offer active sites for oxygen chemisorption, hence enhancing gas sensing efficacy.^49^ Conversely, OH^−^ species on the surface of ferrites may be responsible for O_ads2_.^50^

**Figure 3:**
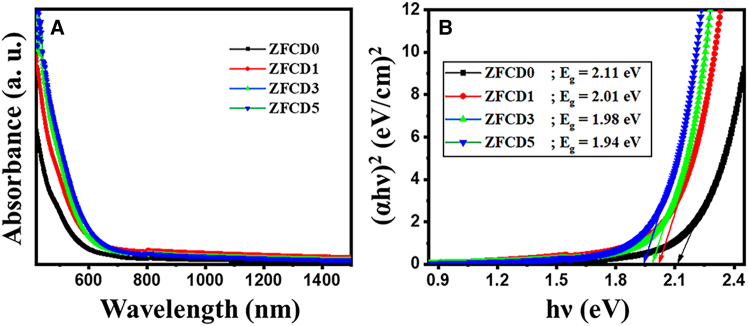
Optical properties of ZFCD0 - ZFCD films (A) UV-Visible absorption spectra. (B) Band gap obtained by Tauc’s plot for ZFCD0, ZFCD1, ZFCD3, and ZFCD5 films.

The absorption spectra of the synthesized films (ZFCD0-ZFCD5), acquired using UV-Vis spectroscopy, are illustrated in Figure 3. Figure 3A illustrates that the absorption edge red-shifts from 580 nm to 640 nm with increasing Cd concentration. This change is ascribed to the inclusion of Cd, which exhibits significant background absorption in the visible-light spectrum and can further augment the light-harvesting capacity of the Cd-doped films.^5^ The band-gap energies were determined utilizing Tauc’s approach, predicated on the relationship:

(Equation 3) $$\alpha h\nu =A\;{\left(h\nu -E_{g}\right)}^{n}$$

where α denotes the absorption coefficient of the material, h signifies the Planck constant, and *ν*indicates the photon frequency, E_g_ is the band gap energy of the semiconductor, whereas the value n represents electronic transitions, specifically *n* = 2 for direct transitions. The band gap values of Cd-doped and undoped films may be determined by graphing (αhν)^2^ vs. hν. The value of the bandgap changes by varying the Cd concentrations, as shown in Figure 3B. The calculated band values are E_g_ = 2.11 eV, 2.01 eV, 1.98 eV, and 1.94 eV for ZFCD0, ZFCD1, ZFCD3, and ZFCD5, respectively. The reduction in band-gap energy with increased Cd content results from alterations in carrier concentration and the emergence of defects that create new energy levels between the valence and conduction bands of ZnFe_2_O_4_. The introduction of Cd dopants creates impurity states that alter the electrical structure: acceptor levels can descend below the original conduction band, whereas donor levels may ascend above the initial valence band.^38^ These modifications diminish the effective band gap. Specifically, doping with Cd^2+^ ions enhances the quantity of charge carriers, resulting in a further decrease in the apparent band-gap energy. M. Shakil et al. and Harita Kumari et al. observed a similar trend that the band-gap energy of zinc ferrite diminishes with rising Cd^2+^ concentration.^24^^,^^42^

**Figure 4:**
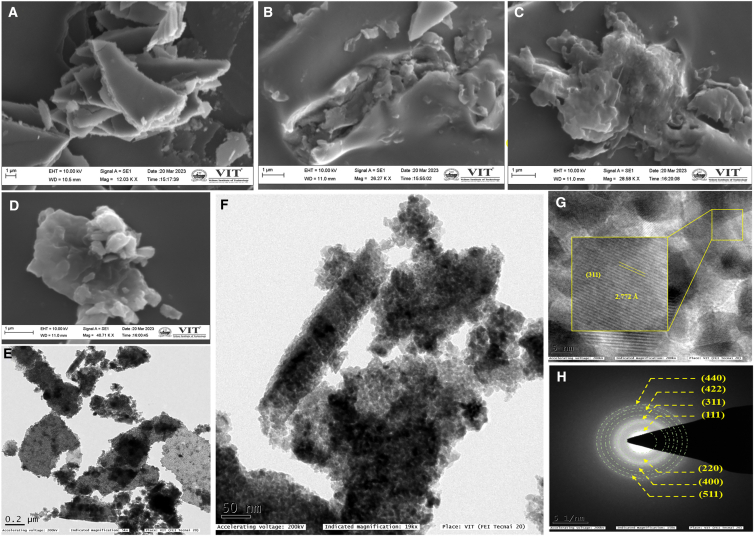
Morphology of the ZFCD0, ZFCD1, ZFCD3, and ZFCD5 films (A–D) SEM image of (A) ZFCD0, (B) ZFCD1, (C) ZFCD3, and (D) ZFCD5 film. Scale bars = 1 μm. (E–H) TEM analysis of the ZFCD5 film: (E) TEM image. Scale bars = 200 nm. (F) magnified TEM image. Scale bars = 50 nm. (G) HRTEM image. Scale bars = 5 nm. (H) SAED pattern. Scale bars = 5 1/nm.

The surface morphology of the ZFCD0, ZFCD1, ZFCD3, and ZFCD5 thin films was examined using SEM. SEM images of all deposited films are given in Figures 4A–4D. The pure ZnFe_2_O_4_ film SEM images (Figure 4A) revealed an uneven angular-shaped morphology, whereas an increase in the Cd dopant concentration, a morphological transformation was observed, where the angular grains gradually evolved into agglomerated angular and then angular shapes along with tiny rod-like structures. Figure 4C shows the angular shapes changed into an agglomerated structure. However, in the case of higher doping concentration, a more angular shape and a tiny rod morphology were observed, as given in Figure 4D. This change in morphology is attributed to the influence of Cd^2+^ incorporation (the greater ionic radius of Cd^2+^ relative to Zn^2+^), which affects nucleation and growth mechanisms during film deposition.^51^ Moreover, the oxygen vacancies at higher Cd^2+^ concentrations provide additional nucleation locations, thereby promoting mixed morphological characteristics.^52^ Rod-like structures provide numerous active sites, which are helpful for gas adsorption/desorption and sensor performance.

Figure S2 (supplemental information) shows the elemental analysis performed using energy-dispersive X-ray spectroscopy (EDX), confirming the existence of Zn, Fe, and O in the pure ZnFe_2_O_4_ thin film. In the Cd-doped films, Cd peaks were also detected, verifying the successful incorporation of Cd into the ZnFe_2_O_4_ matrix. Furthermore, EDX mapping (Figure S3) reveals the even distribution of Zn, Fe, O, and Cd elements across the film surface, ensuring homogeneous doping. TEM and HR-TEM were conducted further to examine the microstructural properties of the ZFCD5 thin films. Figures 4E–4H shows the TEM images of the ZFCD5 thin film, revealing agglomerated angular shapes along with rod-like structures, consistent with the morphology observed in the SEM analysis. The average length and breadth of the rods were measured as 208 nm and 52 nm, respectively, using ImageJ software. The corresponding histograms of rod length and breadth are given in Figure S4. Figure 4G shows the distances between adjacent fringes in the ZFCD5 film, viz. 2.772 Å, corresponding to the (311) plane of ZnFe_2_O_4_. The d-spacing of the pure zinc ferrite film is 2.588 Å, as reported in our previous work.^50^ The interplanar distance increased upon the doping of Cd into the ZFCD0 film, indicating that Cd is incorporated into the ZnFe_2_O_4_ host matrix. Furthermore, the SAED pattern (Figure 4H) exhibited a ring pattern, confirming the polycrystalline nature of the film. The diffraction rings correspond to the characteristic planes (111), (220), (311), (400), (422), (511), and (440) of the cubic spinel ZnFe_2_O_4_ structure, aligning well with the XRD results. The slight broadening of SAED spots in the doped samples suggests minor structural distortions induced by Cd incorporation, supporting the observed peak shifts in XRD analysis. These findings prove the successful incorporation of Cd into ZnFe_2_O_4_ while maintaining the crystalline spinel phase.

**Figure 5:**
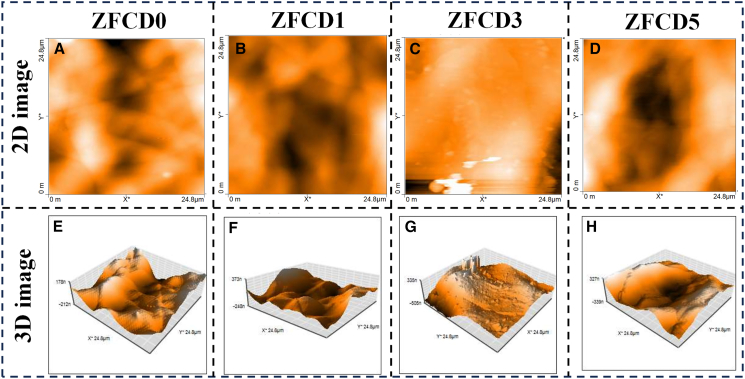
Topography characterization of ZFCD0-ZFCD5 films (A–D) 2D AFM image of (A) ZFCD0, (B) ZFCD1, (C) ZFCD3, and (D) ZFCD5 films. Scale bars = 24.8 μm. (E–H) 3D AFM image of (E) ZFCD0, (F) ZFCD1, (G) ZFCD3, and (H) ZFCD5 films. Scale bars = 24.8 μm.

AFM was employed to examine the topography and roughness of the ZnFe_2_O_4_ and Cd-doped ZnFe_2_O_4_ thin films. AFM images were acquired by scanning a 24.8 × 24.8 μm^2^ region, as given in Figure 5. The image revealed that all thin films exhibited an irregular, rough, minute, crack-free, and closely packed surface morphology. The densely packed grain structure ensures improved stability and uniformity, which are crucial for gas sensing applications. A noticeable increase in the roughness of the surface was noticed with increasing Cd dopant concentration. This increase in roughness can be attributed to the larger ionic radius of Cd^2+^ compared to Zn^2+^, which disrupts the uniform grain growth and introduces lattice strain. Cd alters the nucleation and coalescence processes during film deposition, forming more significant grain boundaries and rougher surfaces. The average roughness and average root-mean-square (RMS) roughness of the prepared films were calculated to be 60, 77, 96, 125 nm and 74, 99, 142, 148 nm, respectively corresponding to ZFCD0, ZFCD1, ZFCD3, and ZFCD5. The roughness value increased with an increase in dopant concentration. The increased roughness enhances the active sites, which is helpful for gas adsorption/desorption and sensor sensitivity.^53^^,^^54^ Additionally, Figure S5 presents the 3D AFM images of the pure and doped films, along with the corresponding height profiles (using gwyddion software) taken along the red dashed line, which reveal the presence of packed surface topography. The films exhibit a certain degree of particle agglomeration, with measured heights of approximately 8 nm, 8.5 nm, 11 nm, and 13 nm for ZFCD0, ZFCD1, ZFCD3, and ZFCD5 films, respectively. These AFM findings further validate the influence of Cd doping on thin film morphology and its potential role in improving gas sensing efficiency.

**Figure 6:**
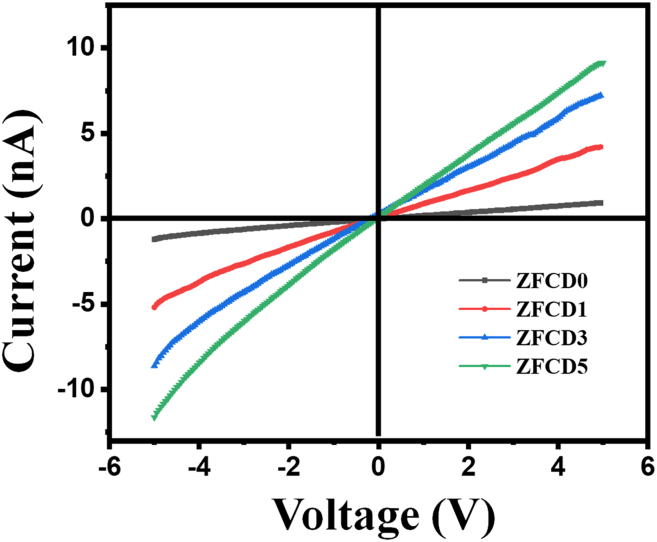
I-V characteristics of the doped and undoped ZnFe_2_O_4_ film

The impact of different Cd concentrations on the sensor’s electrical characteristics has been examined by I-V characteristic measurements. The I-V characteristics curve was obtained by applying a voltage ranging from −5 to 5 V using the Keysight B2901B. Figure 6 illustrates the room temperature current-voltage (I-V) characteristics of sensors with and without Cd in the ZnFe_2_O_4_ layer. The incorporation of Cd markedly affects current conduction. Linear relationships between current and voltage were detected for all devices in darkness, indicating ohmic contacts.^55^ The ZFCD0 sensor has a current of 1.08 nA, whereas the ZFCD5 film exhibits an increased sensor current of 9.18 nA at a forward bias voltage of 5 V. The resistance exhibited the following order: ZFCD0 > ZFCD1 > ZFCD3 > ZFCD5, as given in Figure 6. The augmentation in electrical conductivity is ascribed to the presence of Cd.^56^ This suggests that Cd doping enhances the conductivity of ZnFe_2_O_4_, thereby reducing the depletion width at the metal-semiconductor interface.^57^

### Gas sensing performance

**Figure 7:**
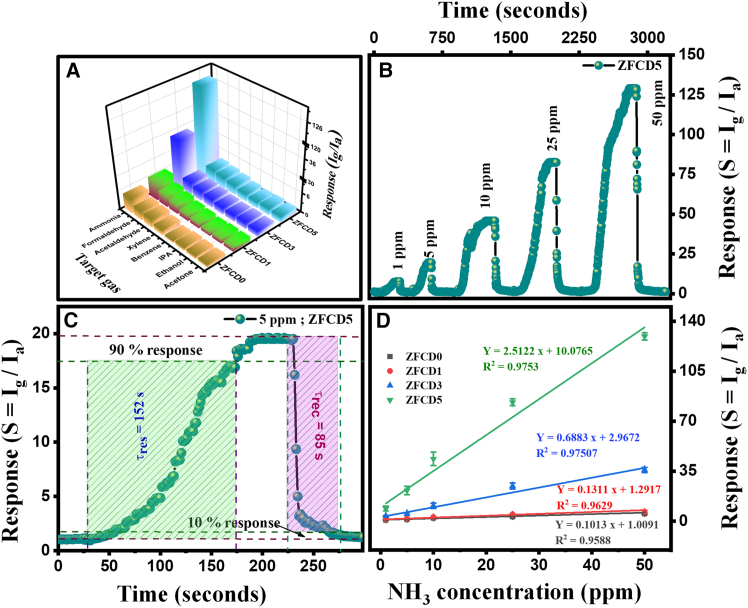
Gas sensing characteristics of pure and doped ZnFe_2_O_4_ films (A) 3D bar diagram of all the sensing film response characteristics toward different gases with a concentration of 50 ppm at room temperature. (B) TRC curves of ZFCD5 toward 1–50 ppm of NH_3_ gas. (C) response/recovery times of ZFCD5 toward 5 ppm of NH_3_ gas. (D) A linear fit between NH_3_ gas concentrations and response values for all the films. Data are represented as mean ± SD (*n* = 3).

The sensing ability of bare and Cd-ZnFe_2_O_4_ films was carefully evaluated utilizing a custom-built gas sensing apparatus. The initial current (baseline) was measured at ambient atmosphere for both the bare and Cd-doped films. The response of the prepared film for vapor detection at room temperature was determined using the equation S = I_g_/I_a_, where I_a_ and I_g_ represent the sensor current measured in ambient and test vapor, respectively. The selectivity of the doped and undoped ZnFe_2_O_4_ thin films was tested against various toxic gases, including acetone, ethanol, isopropanol, xylene, formaldehyde, NH_3_, and acetaldehyde, all at 50 ppm concentration at ambient temperature. Among these gases, ZFCD5 thin film exhibited the highest response (S = 127) toward NH_3_, demonstrating superior selectivity compared to the ZFCD0 (S = 4.6), ZFCD1 (S = 6), and ZFCD3 (S = 34.7) films as given in Figure 7A. The enhanced selectivity of the ZFCD5 film toward NH_3_ gas is that NH_3_ is a reducing gas that donates electrons upon interaction with ZnFe_2_O_4_. The introduction of Cd^2+^ dopants in ZnFe_2_O_4_ increases the structural disorder, making the film more responsive to NH_3_ adsorption. The higher Cd doping level (0.5) optimizes the film’s surface reactivity, improving the gas-sensing response. Additionally, the spinel structure of ZnFe_2_O_4_ and its mixed oxidation states (Fe^2+^/Fe^3+^) facilitate better electron transfer, leading to a strong response toward NH_3_. NH_3_ is a Lewis base gas with a lone pair of electrons on the nitrogen atom, making it prone to connect with acidic sites on the film surface. Cd^2+^ and Fe^3+^ ions create Lewis acid sites that can form coordination bonds with NH_3_ molecules. This strong acid-base interaction leads to the preferential adsorption of NH_3_ over other gases, thereby enhancing selectivity.^58^ Moreover, the energy alignment between the Zn 3d orbitals of ZnFe_2_O_4_ and the N 2pz orbitals of NH_3_ facilitates charge transfer by the direct NH_3_ adsorption onto the sensing elements.^59^

Additionally, a smaller dissociation bond energy (453 kJ/mol) and a smaller molecular size (0.32 nm) of NH_3_ relative to other vapors substantiate the pronounced selectivity of the ZFCD5 angular configuration with diminutive rods. Table 2 enumerates the molecular size and bond strength metrics for each probable interfering gas. A molecule with strong bond strength is difficult to break, whereas a molecule with low bond strength breaks rapidly, perhaps resulting in a faster reaction. In the context of the bond strength of the examined molecules for selectivity in our investigation, the types of bonds present in the target vapors are C=O, N-H, O-H, and C-H, with bond strengths of 748.2, 452, 462, and 748 kJ/mol, respectively. Mingjun Sun et al.^60^ established that NH_3_ had a markedly low and unstable bond energy, facilitating its reaction with the highly catalytic ZFCD5 system, resulting in an enhanced sensing response compared to other vapors.^48^^,^^53^^,^^61^^,^^62^^,^^63^ Cross-sensitivity presents a prevalent challenge in chemiresistive-type gas sensors, as various reducing gases, including ethanol, acetone, formaldehyde, acetaldehyde, and xylene, may interact with the sensing layer. In the current study, the response to these interfering gases was minimal when compared to NH_3_ under the same conditions (Figure 7A). The improved selectivity results from the increased polarity and reduced molecular size of NH_3_, facilitating more efficient adsorption and charge transfer at oxygen-vacancy sites. NH_3_ is a small, highly polar molecule characterized by a lone pair of electrons, significant adsorption properties, and charge transfer interactions with oxygen species on the oxide surface. Volatile organic compounds (VOCs) such as ethanol and acetone are characterized by larger size, reduced polarity, and steric hindrance, leading to diminished adsorption at ambient temperature.^64^ Furthermore, NH_3_ engages with the sensor via robust hydrogen bonding, Lewis base-acid interactions, and electron donation mechanisms, which prevail over the weaker interactions linked to ethanol and isopropanol.^65^ Numerous interfering gases necessitate increased temperatures for dissociative adsorption. At room temperature, the adsorption of NH_3_ is energetically more favorable compared to that of most VOCs. Moreover, operating at room temperature reduces cross-interference from VOCs, which generally necessitate greater activation energy for dissociative adsorption. These factors collectively inhibit cross-sensitivity, leading to a strong preference for NH_3_ detection.

**Table 2:** Molecular size (nm) and bond strength (kJ/mol) of NH_3_ and other test gases

Test gas	Bond strength (kJ/mol)	Molecular size (nm)
Ethanol	462	0.44
Acetone	748	0.66
Isopropanol	462	0.47
Xylene	–	0.45
Formaldehyde	748.2	–
Acetaldehyde	–	0.51
Ammonia	452	0.32
Benzene	518	0.52

The transient response curve (TRC) in Figures 7B and S5A–S5C illustrates that as the NH_3_ gas concentration rises, the sensor response also increases, indicating a direct association between gas absorption and sensor response. From the TRC of the tested films, the ZFCD5 thin film showed the highest response, demonstrating a superior response compared to the pure and lower Cd-doped films. Figure 7B shows that the current value increases as the gas concentration increases from 1 ppm to 50 ppm. The superior gas response of the ZFCD5 film can be attributed to several scientific factors: angular shape, tiny rods, and high roughness (confirmed from SEM and AFM analysis), which provide more active sites for gas adsorption. The angular features characterized by sharp edges and corners create high-energy sites on the film surface that act as active centers for gas adsorption. These defect sites facilitate the adsorption of oxygen species and NH_3_ molecules. The response and recovery times are essential characteristics of chemiresistive sensors. The response and recovery times were significantly improved in the ZFCD5 film, as given in Figure 7C. The observed response time and recovery times are 152 s and 85 s, respectively, implying that the sensor responds quickly to the gas. The ZFCD5 film exhibited a response and recovery time suitable for real-time gas monitoring applications, making it a suitable material for NH_3_ detection. The linear relation amid the NH_3_ response and the target gas concentration is given in Figure 7D, where the different color symbol signifies the definite concentration of NH_3,_ and the line is the straight fitting curve with a high association coefficient of 0.9588, 0.9629, 0.9750, and 0.9753 corresponding to ZFCD0, ZFCD1, ZFCD3, and ZFCD5, respectively representing the linear gas response capability of the film to observe the exact concentration of NH_3_ molecules. Good linearity in the response curve suggests that the sensor follows a well-defined gas adsorption-desorption mechanism, which is essential for practical sensor applications. The trend in Figure 7D confirms that higher Cd doping levels enhance NH_3_ adsorption and improve sensitivity, making the ZFCD5 film an excellent candidate for high-performance gas sensors. The ZFCD5 has a large sensitivity of 10.07 ppm^-1^ and a good linearity of 0.97 compared to ZFCD0 (1 ppm^−1^), ZFCD1 (1.29 ppm^-1^), and ZFCD3 (2.96 ppm^-1^).

**Figure 8:**
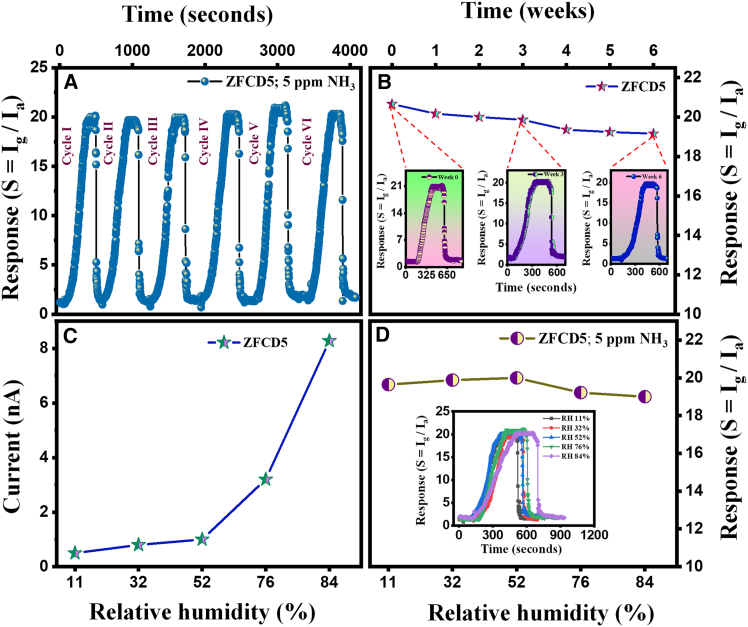
Repeatability, stability, and relative humidity performance of ZFCD5 film (A) Repeatability of ZFCD5 toward 5 ppm of NH_3_ at ambient temperature (six cycles).(B) Prolong stability analysis of ZFCD5 sensor to 5 ppm of NH_3_ (insets: response data of ZFCD5 to NH_3_ at an interval of a week). (C) Baseline current value of ZFCD5 film in different humidity environments. (D) Diverse humidity environment response curve of ZFCD5 film toward 5 ppm NH_3_.

The repeatability of the ZFCD5 film was evaluated using six consecutive sensing cycles at an NH_3_ concentration of 5 ppm, as given in Figure 8A. The sensor maintained a stable response throughout the cycles, with only a slight reduction observed after the 6^th^ cycle. Notably, there was no shift in the baseline current, indicating excellent reproducibility. The relative standard deviation over six cycles was approximately 2%. The slight decrease in response after multiple cycles can be attributed to minor surface saturation caused by residual NH_3_ molecules remaining adsorbed on the film after prolonged exposure. Another contributing factor is the gradual deactivation of active surface sites. Over time, the surface of the sensing film may become contaminated with airborne impurities such as moisture, hydrocarbons, or other environmental pollutants.

These species can occupy or block adsorption sites normally used for gas interaction, thereby reducing the number of available sites for NH_3_ adsorption and ultimately lowering the sensor’s response. However, the consistent baseline current confirms that the sensor retains its original conductivity and does not undergo significant degradation, making it suitable for long-term applications. To evaluate the long-term stability of the ZFCD5 gas sensor, a stability test over six weeks was conducted at room temperature. The sensor was periodically tested with 5 ppm of NH_3_ at regular intervals, and its response was recorded. Figure 8B shows that the ZFCD5 thin film maintained a consistent sensing response throughout the six-week duration, with only a slight decrease from 20.66 to 19.86 in the 4 weeks, but then almost steadies, indicating more stability. The baseline current was assessed weekly over six weeks, demonstrating a gradual rise from 9.748 × 10^−10^ A in week 0–1.1690 × 10^−9^ A in week 6. This represents a 19% increase from week 0 to week 6. A linear regression analysis of baseline versus time, shown in Figure S7 produced a slope of (2.49 ± 1.01) ×10^−11^ A week^−1^ (r = 0.82, *p* = 0.069), indicating no statistically significant drift during the monitoring period. The stability of the sensor can be attributed to the robust crystalline structure of ZnFe_2_O_4_ and the strong bonding of Cd^2+^ ions within the spinel structure, which prevents material degradation in the long time. The impact of relative humidity (RH) on NH_3_ gas sensing response was investigated at different humidity conditions ranging from 11% to 84% RH. RH during gas-sensing experiments was regulated using typical saturated salt solutions positioned within the test chamber. The relative humidity levels were regulated by including a saturated aqueous solution of LiCl, MgCl, MgNO_3_, NaCl, and KCl to maintain consistent humidity values of 11%, 32%, 52%, 76%, and 84% RH in the test chamber.^66^^,^^67^ By selecting suitable salts, the necessary relative humidity settings for the gas reaction experiment were created. The relative humidity (RH%) was recorded using a typical digital hygrometer (HTC Instruments, HD-306, India) connected to the sensing chamber. Figure 8C illustrates that when humidity increases from low (11%) to high (84%) relative humidity, the baseline current of the ZFCD5 film climbs markedly. As humidity increases, physically adsorbed H_2_O molecules will start forming on the film surface of the ZFCD5 film, creating a proton (H^+^) transfer layer. Figure 8D shows that the ZFCD5 film gave a stable and high response to NH_3_ even under varying humidity conditions. Although a slight reduction in response was observed at very high humidity levels (above 80% RH), the overall sensing performance remained efficient. This is because water molecules adsorb onto the sensor surface under highly humid environments, potentially interfering with NH_3_ adsorption. The physically adsorbed H_2_O molecules would take up the active sites on the ZFCD5 sensor, thus avoiding the response between the sensing film and the gas molecules. Moreover, the response intensity exhibited minimal fluctuation, with a coefficient of variation (CV) of 2% (Figure 8B), signifying a robust capacity to endure elevated humidity levels while precisely detecting NH_3_ under these circumstances. Interestingly, the achieved higher sensor response value and low concentration range were significantly lower than the earlier reported sensors, as summarized in Table 3.

**Table 3:** A comparative analysis of NH_3_ sensing investigations between the current research and existing literature

Sensing element	Methods	NH_3_ (ppm)	Response	Operating temperature (°C)	Low concentration detection (ppm)	Reference
ZnO-Cr	Hydrothermal	50	0.5	RT	10	Nakarungsee et al.^68^ 2020
ZnO/rGO	Spray pyrolysis	10	1.20	RT	10	Tai et al.^69^ 2016
Ni-ZnO	Spray pyrolysis	100	2.52	RT	5	Mani and Rayappan^70^ 2014
Co-ZnO	Spray pyrolysis	100	3.48	RT	15	Mani and Rayappan^71^ 2015
Ti/Graphene	Chemical vapor deposition	400	7	RT	12	Zhao et al.^56^ 2017
Pt/Al-ZnO	RF sputtering	1000	24	300	1	Chen et al.^72^ 2018
Fe_2_O_3_	Thermal oxidation	100	18.8	250	5	Lupan et al.^73^ 2017
ZnFe_2_O_4_	Spray pyrolysis	100	30	RT	10	Ravikumar et al.^36^ 2023
Cd-ZnFe_2_O_4_	Spray pyrolysis	5	20	RT	1	This work

### Gas sensing mechanism

**Figure 9:**
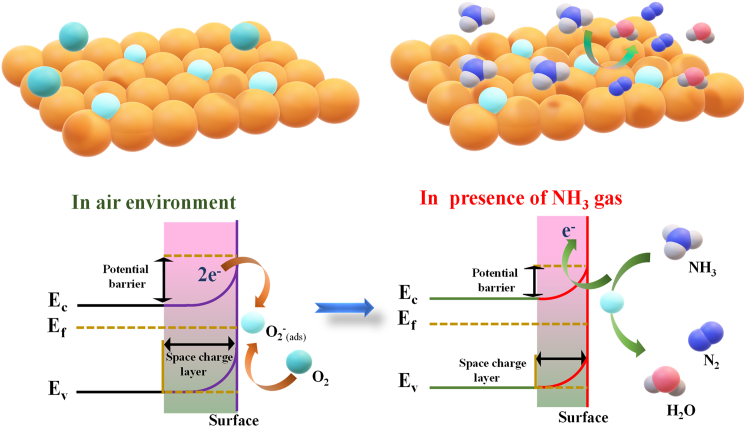
A schematic of the gas sensing mechanism of ZnFe_2_O_4_ thin film, both before and after NH_3_ presence

The principle of gas detection is that the electrical changes of a semiconducting metal oxide vary with the existence or absence of a test gas molecule. The three essential processes in this procedure are absorption, desorption, and charge transfer. The sensing mechanism of Cd-doped ZnFe_2_O_4_ films toward NH_3_ follows a typical chemiresistive gas sensing process, primarily governed by the interaction between surface-adsorbed oxygen species and the target gas. When the sensor is exposed to ambient air, oxygen molecules O_2_ are adsorbed onto the film surface and capture delocalized electrons from the conduction band of ZnFe_2_O_4_, creating negatively charged oxygen such as O^−^and O_2_^−^. This procedure leads to the formation of a depletion layer at the surface, increasing the electrical resistance of the film. Upon exposure to NH_3_, which is a reducing gas, electron-donating reactions occur, where NH_3_ molecules react with the surface-adsorbed oxygen species, releasing electrons back into the conduction band so that the depletion layer thickness gets reduced, as given in Figure 9. The reaction can be expressed as follows:

(Equation 4) $${2\text{NH}}_{3}+O_{2\left(\text{ads}\right)}^{-}\;\to \;N_{2}+{3H}_{2}O+{3e}^{-}$$

NH_3_ is a reducing gas that reacts with chemisorbed oxygen species and easily oxidizes to N_2_, H_2_O, and electrons.^74^ Electrons are released into the conduction band, causing resistance to drop. This electron reinjection reduces the depletion layer width, resulting in a decrease in resistance, which is detected as a measurable response.

The enhanced sensing performance of ZFCD5 films can be attributed to several factors.

(1) The enhanced adsorption processes and dissociation of adsorbed oxygen and/or target gas at ambient temperature, which facilitate gas diffusion through the small grains of the ZFCD5 nanorod, may account for this improvement. Angular shapes, including tiny nanorod sensors, facilitate supplementary pathways for electron exchange during gas interaction; these sensors consist of a smaller rod size and measure around 208 nm in length and 52 nm in breadth. (2) The observed crystallite sizes between 27 and 22 nm in bare and Cd-doped ZnFe_2_O_4_ films signify a marginal reduction in crystallite size with the addition of Cd. This reduction leads to an expansion in surface area. The increased surface area facilitates greater interaction between gas molecules and the sensor material, resulting in enhanced sensitivity and expedited response times.^75^ The reduced crystallite size, seen in ZFCD5 (22 nm), leads to an increased ratio of the electron depletion layer relative to the total crystallite volume. As a result, nanorods demonstrate a significant alteration in depletion layer thickness and an enhanced gas sensitivity when exposed to NH_3_ gas.^57^ Moreover, the (3) XPS measurements indicate that Cd-doping generates oxygen vacancies in the ZFCD5 layer, hence augmenting the quantity of adsorption sites accessible for ambient oxygen molecules. (4) The ZFCD5 films have a substantially rougher surface morphology compared to the other samples, as verified by AFM and SEM investigations. The augmented surface roughness offers a greater quantity of active sites, thereby promoting more effective interactions with gas molecules and improving overall sensing effectiveness.^76^ Upon removal of NH_3_, O_2_ molecules from the ambient environment re-adsorb onto the surface, restoring the depletion layer and returning the sensor to its baseline resistance. This reversible adsorption-desorption process ensures the repeatability and stability of the sensor, making ZFCD5 a highly efficient material for NH_3_ detection.

## Discussion

In summary, the incorporation of cadmium into ZnFe_2_O_4_ thin films deposited through the spray pyrolysis technique significantly enhanced the structural, morphological, and gas sensing properties of the material. XRD analysis confirmed that Cd doping induces lattice expansion. Raman spectroscopy further validated the integrity of the spinel structure despite the structural modifications introduced by Cd. The distinctive angular grains and rod-like morphology observed through SEM, TEM, and AFM analyses contributed to an increased surface area, facilitating rapid diffusion and adsorption of NH_3_ molecules. The ZFCD5 gas sensor demonstrates exceptional efficacy in detecting trace levels of NH_3_ at room temperature, characterized by a good response (S = 8 at 1 ppm), sensitivity (10.07 ppm^-1^), remarkable selectivity, along with excellent repeatability, surpassing all previously reported pure ZFCD0 film and the majority of ZFCD5 counterparts. These findings highlight the latent of Cd-doped ZnFe_2_O_4_ films as effective and reliable gas sensors for environmental and real-time industrial monitoring applications. Moreover, the development of reliable, sensitive ammonia sensors can play an important role in protecting individuals with disabilities or health vulnerabilities by providing early warning in situations where personal detection may be limited.

### Limitations of the study

Our investigation reveals encouraging low-concentration NH_3_ detection and strong sensor performance over a six-week duration; nonetheless, some limitations persist. The long-term stability beyond six weeks has not been assessed; Ternary metal oxide sensors often demonstrate baseline drift, baseline change, and sensitivity deterioration over prolonged durations or fluctuating environmental conditions. Secondly, our spray pyrolysis fabrication method, while dependable at the laboratory scale, has difficulties in scaling to commercial production, including the maintenance of uniform morphology, precursor use efficiency, and repeatability under high-throughput settings. Ultimately, the sensor demonstrated remarkable repeatability and selectivity under diverse humidity conditions; its resilience to broader environmental stresses, such as extreme temperature variations, interference from mixed gases, and mechanical disturbances, has yet to be evaluated; these factors are recognized to considerably affect metal-oxide sensor stability and necessitate compensation strategies or design enhancements.

## Resource availability

### Lead contact

Further information and other requests should be directed to and will be fulfilled by the lead contact, Andrews Nirmala Grace (anirmalagladys@gmail.com).

### Materials availability

This work did not generate new unique reagents. All the materials and methods used for the generation of data and analysis are mentioned in the article.

### Data and code availability

All data reported in this article will be shared by the lead contact upon request. This article does not report original code. Any additional information required to reanalyze the data reported in this article is available from the lead contact upon request.

## Acknowledgments

The authors express gratitude to the Council of Scientific and Industrial Research (CSIR), New Delhi, India, for financial assistance via the Government of India for project fellowship [File No: 09/0844(18460)/2024-EMR-I], and to VIT administration for the provision of infrastructural facilities. The authors extend their appreciation to the King Salman Center for Disability Research for funding this work through Research Group no KSRG-2024-382. This research work was supported by the Anusandhan National Research Foundation (ANRF) under the Partnerships for Accelerated Innovation and Research (PAIR) project, Government of India, sanction order ANRF/PAIR/2025/000011/PAIR-B.

## Author contributions

Thangavel Ravikumar: methodology, investigation, formal analysis, writing – original draft, and writing – review and editing. Kalainathan Sivaperuman: conceptualization, investigation, validation, and writing – review and editing. Logu Thirumalaisamy: methodology, validation, and formal analysis. Christina Josephine Malathi A: resources and formal analysis. Saravanan Pandiaraj: formal analysis Maha Alruwaili: validation and formal analysis. Nadyah Alanazi: visualization, formal analysis, and funding acquisition Abdullah N Alodhayb: visualization, validation, and formal analysis R. Ramesh: formal analysis. Chamil Abeykoon: formal analysis Andrews Nirmala Grace: conceptualization, funding acquisition, validation, formal analysis, supervision, and writing – review and editing.

## Declaration of interests

The authors declare no competing interests.

## STAR★Methods

### Key resources table

**Table:** 

REAGENT or RESOURCE	SOURCE	IDENTIFIER
**Chemicals, peptides, and recombinant proteins**		

Zinc nitrate hexahydrate	Sigma Aldrich	CAS: 10196186
Iron nitrate nonahydrate	Sigma Aldrich	CAS: 7782618
Cadmium nitrate tetrahydrate	Sigma Aldrich	CAS: 10022681
Acetone	Sigma Aldrich	CAS: 67641
Acetaldehyde	Sigma Aldrich	CAS: 75070
Formaldehyde	Sigma Aldrich	CAS: 50000
Ammonia	Sigma Aldrich	CAS: 7662217
Benzene	Sigma Aldrich	CAS: 71432
Ethanol	Alpha Scientific	CAS: 64175
Xylene	Sigma Aldrich	CAS:1330207
2-propanol	Sigma Aldrich	CAS: 67630

**Software and algorithms**		

Origin	2018	https://www.originlab.com
ImageJ	1.53a	https://www.imagej.net/ij/
Gwyddion	2.66	https://gwyddion.net

**Other**		

X-ray diffraction	PANalytical	PANalytical Xpert pro
Uv-visible spectroscopy	Agilent	Agilent Carry 5000
Raman spectroscopy	HORIBA	HORIBA XPLORA plus
X-ray photoelectron spectroscopy	Thermo Fisher Scientific	Thermo Fisher Scientific K-alpha
Scanning electron microscopy	Zeiss	Zeiss EVO/18
Transmission electron microscopy	FEI-TECHAI	FEI-TECHAI G2-20 Twin
Atomic force microscopy	Nanosurf	Nanosurf EASY scan-2
I-V measurement	Keysight	Keysight B2901B
Gas sensing measurement	Keithley	Keithley – 6517B

### Experimental modeland study participant details

There are no experimental models (animals, human participants, plants, microbe strains, cell lines, primary cell cultures) used in the study.

### Method details

#### Thin film deposition

ZnFe_2_O_4_ and Cd-ZnFe_2_O_4_ thin films were synthesized using the chemical spray pyrolysis (CSP) method, a cost-effective and scalable technique for fabricating high-quality TMFs thin films. In this process, precursor solutions of zinc nitrate (Zn(NO_3_)_2_·6H_2_O) and ferric nitrate (Fe(NO_3_)_3_·9H_2_O) were dissolved in double distilled water (as given in Figure S1A), with controlled molar ratios of 1:2. For cadmium doping, cadmium nitrate (Cd(NO_3_)_2_·4H_2_O) was added to the precursor solution at varying doping concentrations (x = 0, 0.1, 0.3, and 0.5) to investigate its effect on the thin film properties. The prepared solution was then atomized into tiny droplets using a spray nozzle and was made to fall onto a preheated substrate, usually soda lime glass, maintained at an optimized substrate temperature at 225 °C, as illustrated in Figure S1B. Our earlier work detailed the comprehensive examination of substrate temperature, revealing that 225 °C yielded optimal crystallinity, surface shape, and adhesion^36^ and hence 225°C was chosen as the best temperature for this investigation. The detailed spray deposition parameters are given in the supplemental information table (Table S1). The high temperature facilitated the decomposition of precursor salts, leading to the formation of a uniform, adherent ZnFe_2_O_4_ and Cd_x_Zn_1-x_Fe_2_O_4_ thin film and the deposited film is named dependent on the dopant concentration (x = 0, 0.1, 0.3 and 0.5) as ZFCD0, ZFCD1, ZFCD3, and ZFCD5 respectively.

#### Characterization

All deposited films are characterized by various characterization methods. XRD (X-ray diffraction), with X-rays of Cu-Kα (λ = 1.5406 Å) by the PANalytical Xpert Pro) was employed to determine the crystallinity and phase structure of the films, confirming the formation of the spinel ZnFe_2_O_4_ structure and analyze the structural modifications occurred due to Cd doping. SEM (scanning electron microscopy, Zeiss EVO/18 Research), TEM (transmission electron microscopy, FEI-TECHAI G2-20 Twin, USA) and AFM (atomic force microscopy, Nanosurf EASY scan-2, Switzerland) were utilized to investigate the surface morphology, roughness, and film uniformity. EDX (Energy-dispersive X-ray spectroscopy) was used to check the elemental composition and verify the successful incorporation of Cd dopants. Raman spectroscopy (HORIBA Scientific XPLORA Plus, France) provided insights into the vibrational modes and chemical bonding characteristics of the deposited films. Optical characteristics of doped and undoped ZnFe_2_O_4_ film were studied by using UV-Visible spectrophotometer (Agilent carry 5000, USA). Aluminum K-alpha micro-focused monochromator (Thermo Fisher Scientific, USA) was used to acquire XPS (X-ray photoelectron spectroscopy) spectra. Current-voltage (I-V) characteristics of deposited films was obtained by keysight B2901B, Malaysia.

#### Gas sensing measurement

The gas sensing characteristics of ZnFe_2_O_4_ and Cd-doped ZnFe_2_O_4_ thin films was evaluated using a custom-built gas sensing setup. Initially, the coated films were cut into 15 × 15 mm^2^ pieces. On the coated surface, two parallel electrodes (2 mm width) were fabricated using conductive silver paste applied with a brush. The distance between the two electrodes was maintained at 11 mm, as illustrated in Figure S1C. These electrodes were then linked to an electrometer (Keithley- 6517B) using a two-probe setup with a bias voltage of 5 V for the sensors, which was interfaced with a computer for data acquisition. The sensing film was placed inside the gas sensing chamber with a capacity of 1 L, and the sensor current was continuously monitored until it reached a stable baseline value, as shown in Figure S1D. Once a stable baseline was achieved, a desired concentration of test gas was introduced into the chamber. The target vapor concentration was ascertained utilizing the static liquid-gas distribution technique.^54^ The target gas solutions were acquired commercially, with the following specifications: acetone (Sigma-Aldrich, ≥99.5%, density = 784 kg/m^3^), acetaldehyde (Sigma-Aldrich, ≥99.5%, density = 788 kg/m^3^), formaldehyde (Sigma-Aldrich, 36.5–38% in H_2_O, density = 1.09 g/mL), ammonia (Sigma-Aldrich, 30–33% NH_3_ in H_2_O, density = 0.9 g/mL), benzene (Sigma-Aldrich, ≥99.9%, density = 0.8787 g/cm^3^), ethanol (Alpha Scientific, 99.9%, density = 789 kg/m^3^), xylene (Sigma-Aldrich, ≥99%, density = 0.859 g/mL), and 2-propanol (Sigma-Aldrich, ≥70% in H_2_O). The gas concentration was determined using the following formula

(Equation 5) $$C_{\text{ppm}}=\frac{\;V_{\tau }*\;\delta *T*R*10^{6}}{P_{b}*V_{b}*\text{MW}}$$

where V_τ_,δ, T, R, P_b_, V_b_, and MWare the volume of the test gas within the testing chamber, density of the test gas, absolute temperature, universal gas constant, chamber pressure, the volume of the chamber, and molecular weight of test gas, respectively. Upon exposure to the test gas, the sensor attained its maximum response, after which the system stabilized. Following the venting of the gas, the sensor returned to its initial baseline state. The sensor response was evaluated as the change in electric current upon gas exposure, which was attributed to the adsorption and desorption of gas molecules on the film’s surface. The response time (time required to reach maximum 90% of the response) and the recovery time (time required to back to the baseline value) were analyzed to assess sensor efficiency. Additionally, the effect of Cd doping on sensitivity, selectivity, and response kinetics was thoroughly investigated. All gas sensing tests were measured at room temperature (300 K).

### Quantification and statistical analysis

Statistical analysis of data was performed using Excel (Microsoft) and Origin (Origin Lab).
