# Supplementary material for: Trace level detection of NH3 at room temperature using Cd-ZnFe2O4 thin films
Source: iScience. 2025 Nov 29;29(1):114271. doi: 10.1016/j.isci.2025.114271 (PMC12767870; doi:10.1016/j.isci.2025.114271)
Supplement: Document S1. Figures S1–S7 and Table S1 [file mmc1.pdf]

iScience, Volume *29*

Supplemental information

Trace level detection of NH_3 at room temperature using Cd-$ZnFe_2O_4$ thin films

Ravikumar Thangavel, Kalainathan Sivaperuman, Logu Thirumalaisamy, Christina Josephine Malathi A, Saravanan Pandiaraj, Maha Alruwaili, Nadyah Alanazi, Abdullah N. Alodhayb, R. Ramesh, Chamil Abeykoon, and Andrews Nirmala Grace

Supplemental information

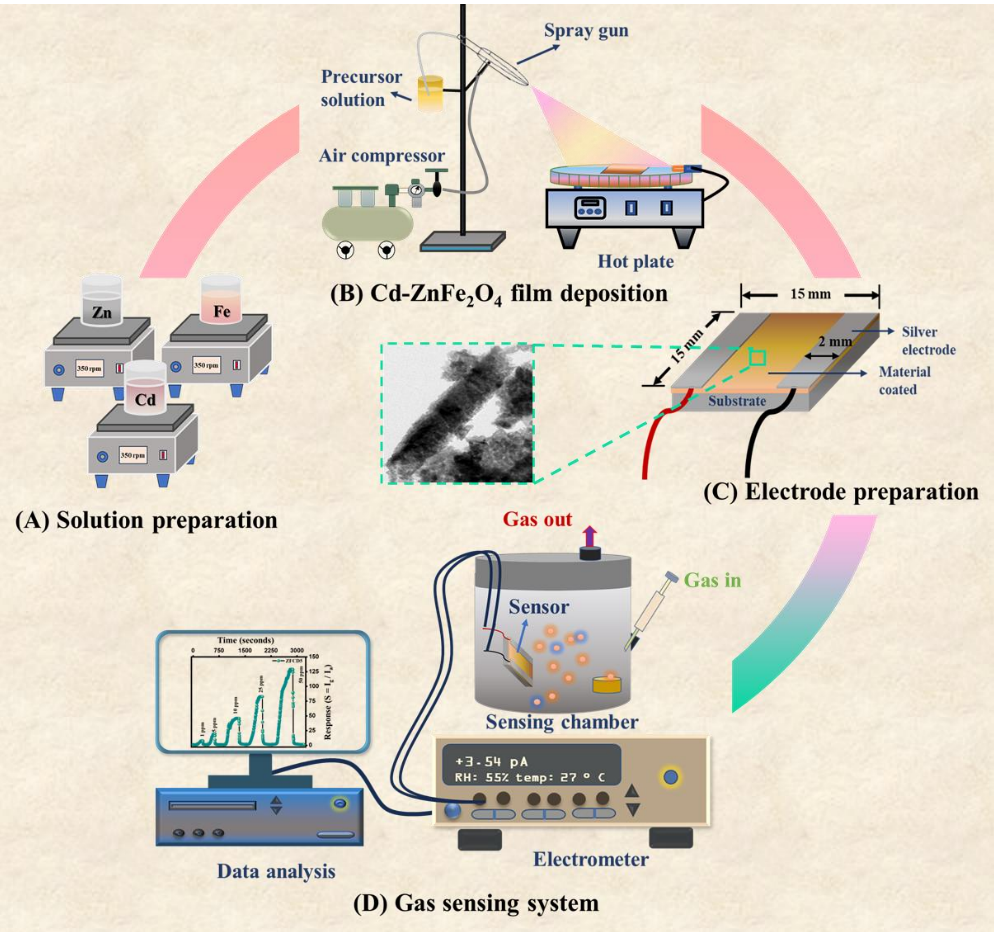

Figure S1. Schematic of Cd–$ZnFe_2O_3$ films fabrication and gas sensing setup.

(A) solution preparation

(B) Cd-$ZnFe_2O_4$ film spray deposition

(C) deposited film electrode preparation

(D) gas sensing measurement setup.

Table S1: Spray parameters employed in film deposition via the chemical spray pyrolysis technique.

**Spray deposition parameters**	**Optimum value**
Precursor molarity	0.05 M
Substrate temperature	225 °C
Solvent	Deionized water
Nozzle- substrate distance	31 cm
Volume of the solution	40 mL
Spray rate	3 mL/min
Carrier gas / atmosphere	Compressed air / ambient
Number of cycles coated	35
Spray angle (degree)	40

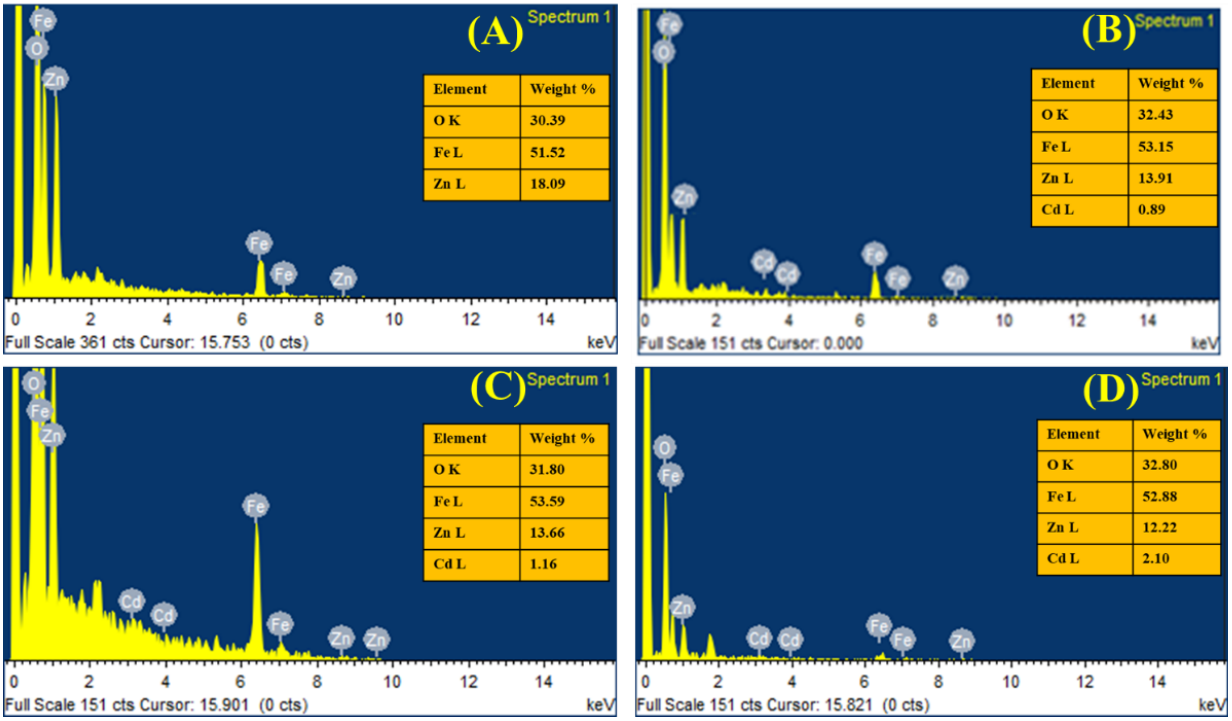

Figure S2. EDX spectra of pure and Cd-$ZnFe_2O_4$ films.

EDX spectra of (A) ZFCD0 (B) ZFCD1, (C) ZFCD3, and (D) ZFCD5 thin films, Related to Figure 4.

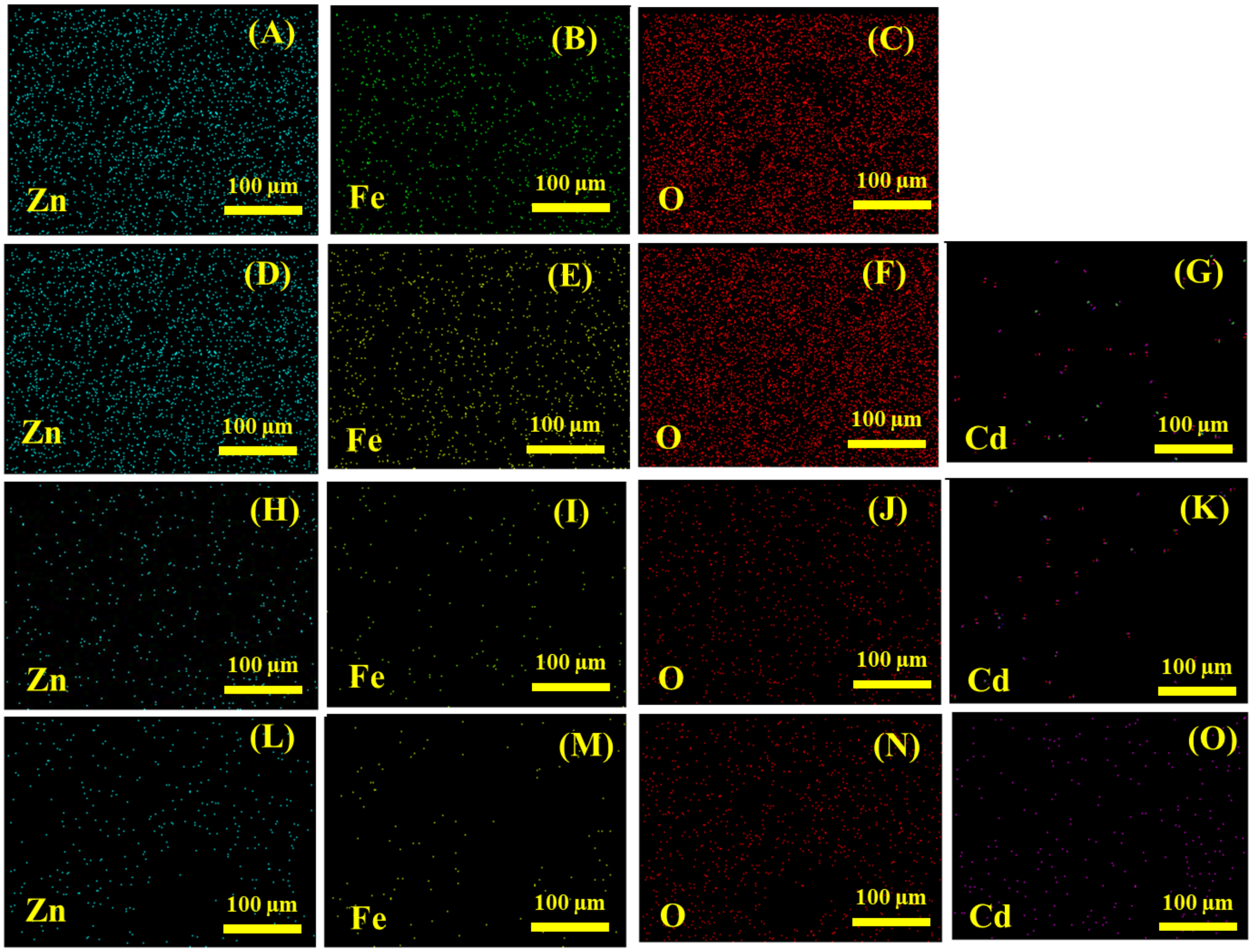

Figure S3. Elemental mapping of pure and Cd-$ZnFe_2O_4$ films.

Elemental mapping of ZFCD0 (A-C), ZFCD1 (D-G), ZFCD3 (H-K), and ZFCD5 (L-O), Related to Figure 4. Scale bar = 100 µm.

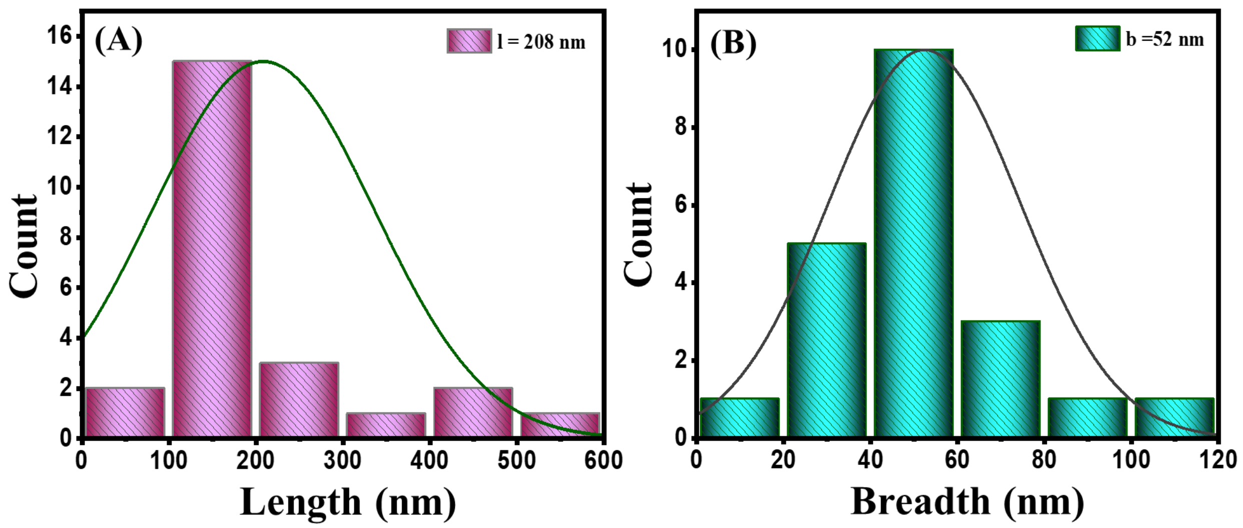

Figure S4. Size distribution analysis of ZFCD5 film.

(A) Length and (B) breadth histogram distribution of ZFCD5 film.

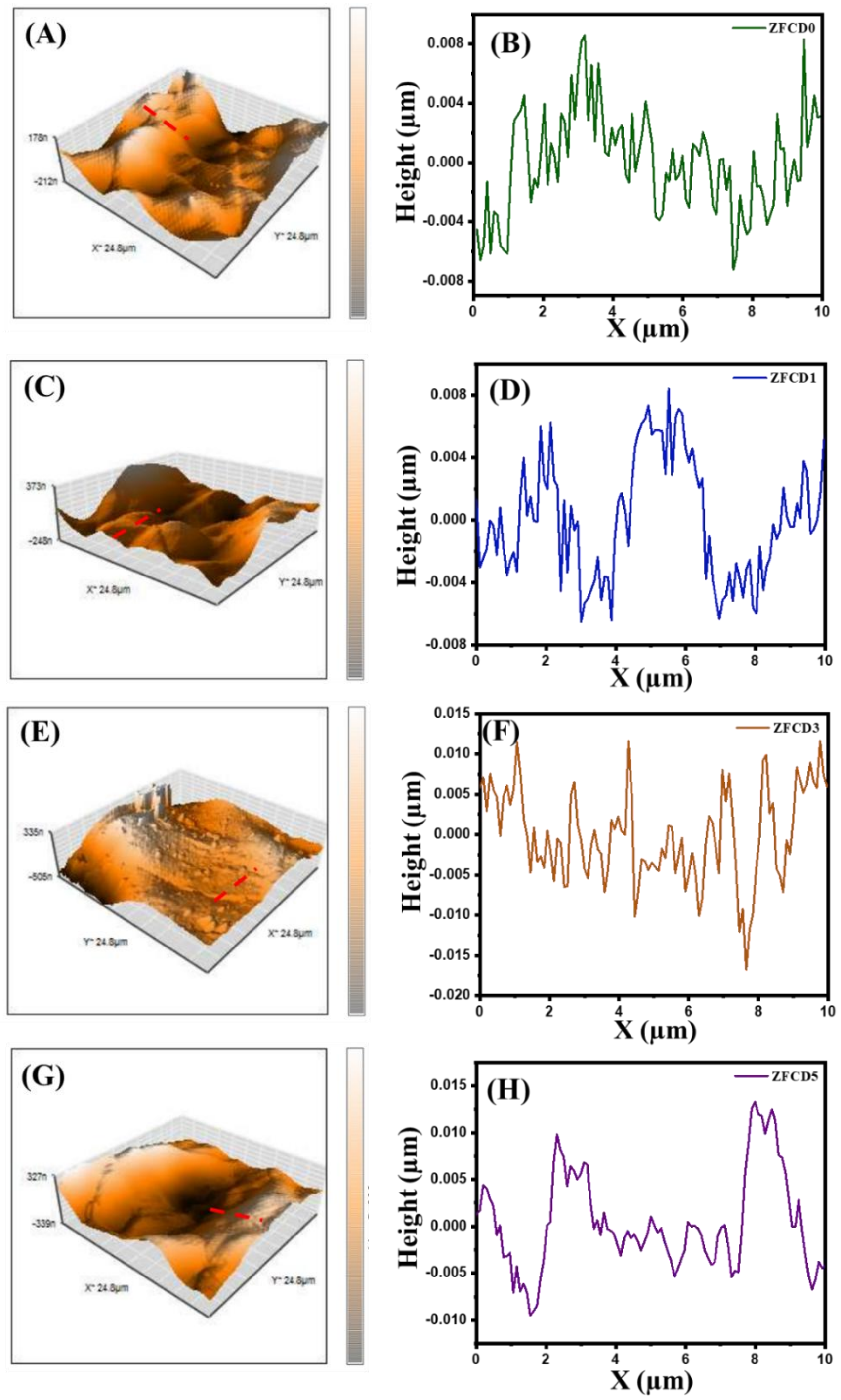

Figure S5. Three-dimensional AFM topography and height profile of pure and Cd-$ZnFe_2O_4$ films.

3D AFM images and height profile plots with red lines for (A, B) ZFCD0, (C, D) ZFCD1, (E, F) ZFCD3, and (G, H) ZFCD5, Related to Figure 5. Scale bar = 24.8 µm.

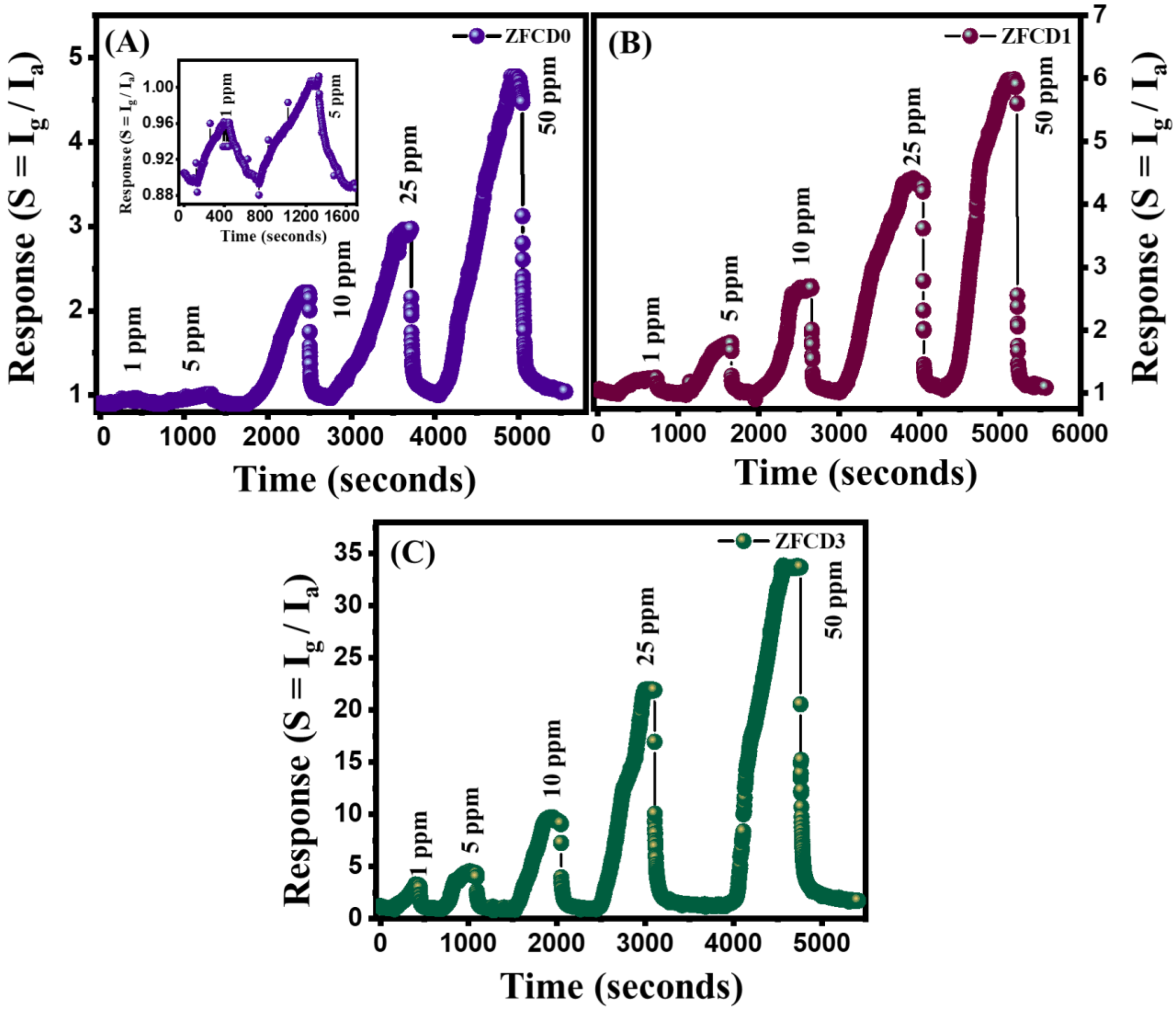

Figure S6. Transient response curve of pure and Cd-$ZnFe_2O_4$ film towards NH_3 gas.

TRC curves of (A) ZFCD0, (B) ZFCD1, and (C) ZFCD3 film towards 1- 50 ppm of NH_3 gas, Related to Figure 7.

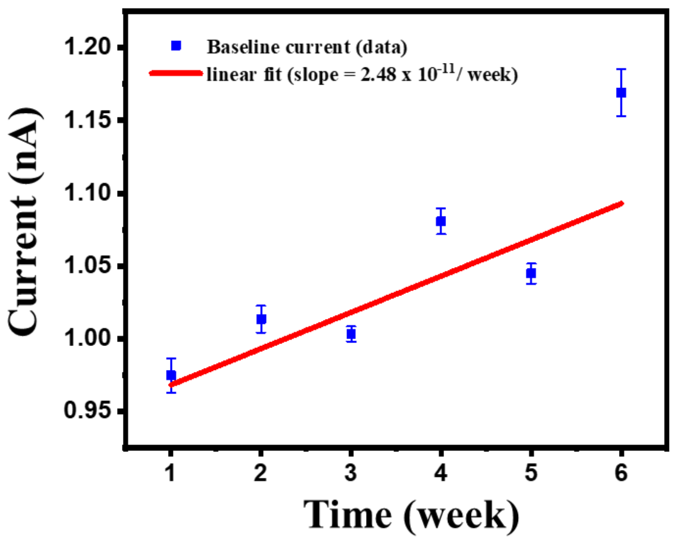

Figure S7. Baseline current stability of ZFCD5 film over time periods.

Linear fit of ZFCD5 film time vs baseline current, data are represented as mean ± SD (n=3), Related to Figure 8.
